# Theta power and theta‐gamma coupling support long‐term spatial memory retrieval

**DOI:** 10.1002/hipo.23284

**Published:** 2020-12-02

**Authors:** Umesh Vivekananda, Daniel Bush, James A. Bisby, Sallie Baxendale, Roman Rodionov, Beate Diehl, Fahmida A. Chowdhury, Andrew W. McEvoy, Anna Miserocchi, Matthew C. Walker, Neil Burgess

**Affiliations:** ^1^ Department of Clinical and Experimental Epilepsy UCL Queen Square Institute of Neurology London UK; ^2^ UCL Institute of Cognitive Neuroscience London UK; ^3^ Wellcome Centre for Human NeuroImaging University College London London UK

**Keywords:** gamma, hippocampus, phase amplitude coupling, spatial memory, theta

## Abstract

Hippocampal theta oscillations have been implicated in spatial memory function in both rodents and humans. What is less clear is how hippocampal theta interacts with higher frequency oscillations to support long‐term memory. Here we asked 10 presurgical epilepsy patients undergoing intracranial EEG recording to perform a long‐term spatial memory task in desktop virtual reality and found that increased theta power in two discrete bands (“low” 2‐5 Hz and “high” 6‐11 Hz) during cued retrieval was associated with improved task performance. Similarly, increased coupling between “low” theta phase and gamma amplitude during the same period was associated with improved task performance. Finally, low and high gamma amplitude appeared to peak at different phases of the theta cycle; providing a novel connection between human hippocampal function and rodent data. These results help to elucidate the role of theta oscillations and theta‐gamma phase‐amplitude coupling in human long‐term memory.

## 1 INTRODUCTION

Oscillations in the local field potential (LFP) reflect synchronous neural activity and are a likely candidate to integrate functional brain regions across multiple spatiotemporal scales (Fries et al., 2007; Buzsáki and Schomburg, 2015). In particular, oscillations within the hippocampal‐entorhinal system have long been hypothesized to play a role in cognitive function. The theta rhythm has been well documented in the rodent and human hippocampal network during translational movement and memory function (Vanderwolf, 1969; O'Keefe and Nadel, 1978; Düzel et al., 2010; Buzsáki and Moser, 2013). Theta frequency in rodents is typically 6–12 Hz, but in the human hippocampus, theta frequency appears to be lower and occupy discrete “low” (2–5 Hz) and “high” (6–11 Hz) frequency bands (Lega et al., 2012; Watrous et al., 2013; Bush et al., 2017). The modulation of higher‐frequency gamma activity by the phase of lower‐frequency oscillations such as theta, manifesting as phase amplitude coupling (PAC), may provide a mechanism for interareal communication and phase coding (Canolty et al., 2006). PAC has been well documented in both human and animal studies during spatial (Tort et al., 2008; Lisman and Jensen, 2013; Newman et al., 2013; Bieri et al., 2014; Tamura et al., 2017), declarative (Fell et al., 2003; Tort et al., 2009; Axmacher et al., 2010; Lega et al., 2016), and sequence memory tasks (Heusser et al., 2016). In particular, the modulation of low (35–50 Hz) and high (60–110 Hz) gamma power by theta phase has been described in both the rodent (Colgin et al., 2009; Colgin, 2015) and human brain for short‐term memory tasks (Alekseichuk et al., 2016; Lega et al., 2016) and episodic recall (Griffiths et al., 2019). However, the presence and purported role of PAC in long‐term spatial memory is uncertain. Here, we characterised the role of low and high theta oscillations, and their relationship with concurrent gamma power, in human intracranial EEG recordings during a self‐paced spatial memory task. We found that low and high theta and low gamma power were significantly increased during spatial memory retrieval and not related to a broadband spectral “tilt” or “shift”. Moreover, increased theta power correlated with successful memory retrieval, and increased PAC between low theta and both low and high gamma oscillations in the hippocampus during retrieval also correlated with task performance. Intriguingly, low and high gamma power peaked at different phases of low theta oscillations, consistent with animal studies (Colgin et al., 2009). These results support the hypothesis that theta‐gamma PAC within the hippocampal formation contributes to successful long‐term memory retrieval in humans.

## 2 METHODS

*iEEG Recordings*. Thirteen patients with drug refractory epilepsy undergoing intracranial EEG monitoring for clinical purposes were asked to perform a long‐term spatial memory task. Prior approval was granted by the NHS Research Ethics Committee (15/LO/1783), and informed written consent was obtained from each subject. Postimplantation CT and/or MRI scans were used to visually inspect and identify electrode locations, confirming that 10 patients had a total of 45 hippocampal electrode contacts (patient demographics are listed in Table 1). Depth EEG was recorded continuously at a sample rate of 1,024 Hz (Patients 4 and 10) or 512 Hz (all other patients) using either a NicoletOne long‐term monitoring system (Natus Medical, Inc.) (Patients 1–6) or Micromed *SD* long‐term monitoring system (Micromed) (Patients 7–10). Recordings made at a higher sampling rate were down‐sampled to 512 Hz, to match those from the majority of patients, before any analyses were performed. All data analyses were performed using the FieldTrip toolbox (Donders Institute for Brain, Cognition and Behaviour, Radboud University, the Netherlands. See http://fieldtriptoolbox.org) (Oostenveld et al., 2011) and custom MATLAB scripts.

**TABLE 1:** Demographics and epilepsy history of patients for spatial memory task

Patient	Age	Gender	Years with epilepsy	Handed	Side of seizure focus	Region of seizure focus	Number of hippocampal contacts ant post		Number of task blocks completed/ mean trials rejected per contact
1	21	F	11	R	R	Temporal	3	1	1/13.25
2	44	F	21	R	R	Temporal	2	3	2/4.8
3	31	M	14	R	L	Temporal	2	2	1/1.5
4	41	M	16	L	R	Temporal	3	2	1/9.4
5	26	M	13	R	R	Temporal	3	2	2/11.8
6	21	M	11	R	L	Occipital	0	2	2/19.5
7	37	F	16	R	R	Temporal	3	1	2/11.75
8	30	F	6	R	B	Temporal	6	2	2/4.5
9	20	F	14	L	R	Temporal	3	2	2/12.2
10	28	M	20	R	R	Temporo‐occipital	3	2	1/8

Abbreviations: F, female; M, male; R, right; L, left; B, bilateral; Ant, anterior; Post, posterior.

**FIGURE 1:**
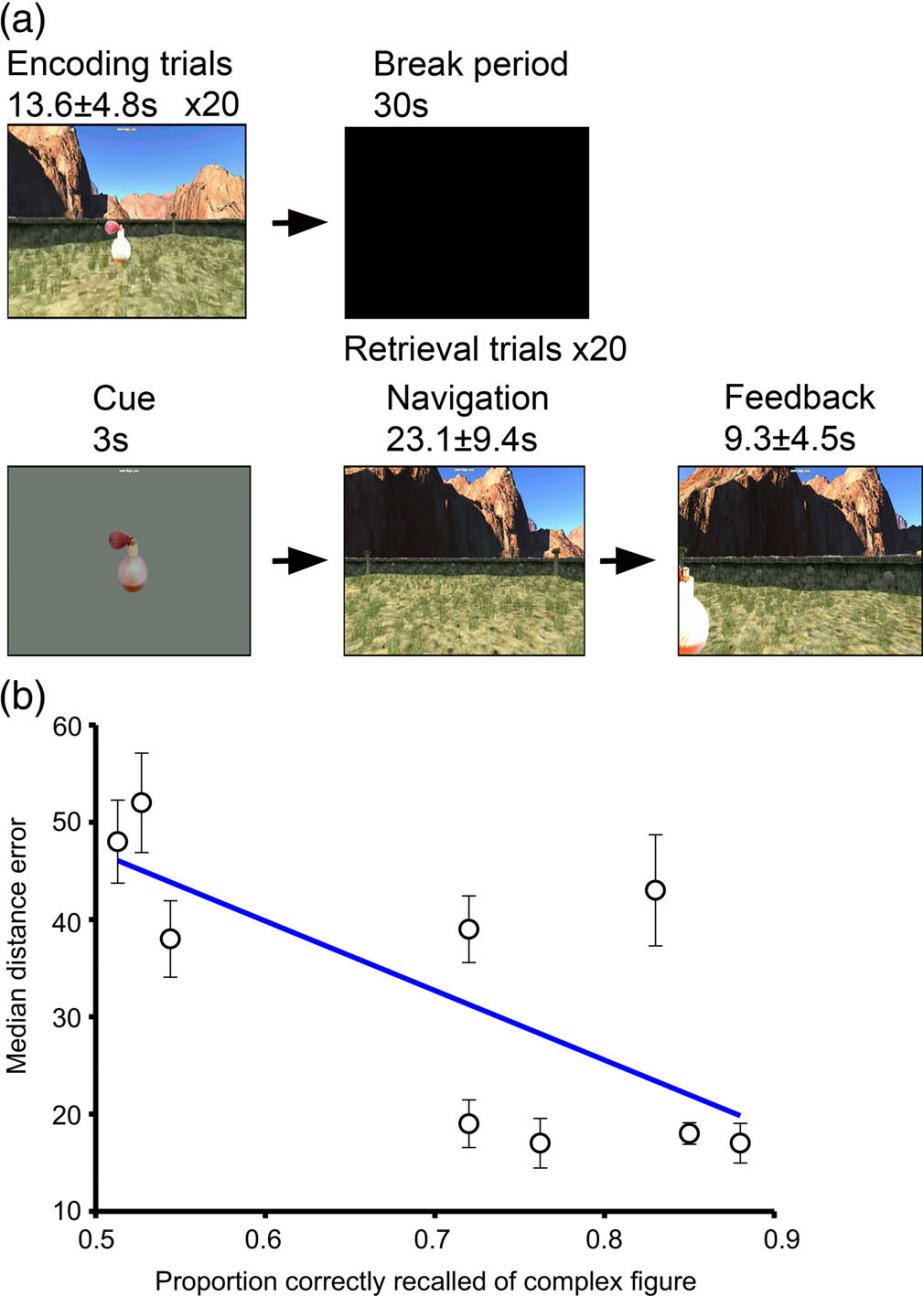
Schematic of long spatial memory task and comparison with clinical neuropsychometry (a). Schematic of the spatial memory task (b). Median distance error (with SEM error bars) in the spatial memory task versus assessment of complex figure recall. Line of best fit shown in blue [Color figure can be viewed at wileyonlinelibrary.com]

*Task*: Long‐term spatial memory was assessed using an “object location task” within a desktop virtual reality environment (Figure. 1a). Patients first navigated toward and memorized the constant location of four objects that appeared in the environment (“encoding”). Encoding comprised of five mini‐blocks, with the four objects appearing sequentially in a random order within each mini‐block, making a total of 20 trials. There was then a 30 s break before the retrieval phase, to ensure that the task assessed long‐term memory. As part of the retrieval phase, patients were cued with an image of one object for 3 s (“cue”), placed back at a random position and orientation within the environment (in order to avoid the use of a stimulus–response strategy) and asked to navigate toward the remembered location of that object and make a button‐press response (“response”). The object then appeared in its correct location and the trial ended when the patient moved to the now visible object (“feedback”). The next trial would begin with the cue screen for another object, before the patient was replaced at another random location and orientation within the same environment. Again there were five mini‐blocks, each with four trials, making a total of 20 trials. Each patient may have completed the same task more than once, in which case, a completely different environment, with different object identities and locations were used. Performance contrasts were computed by taking the median distance error across all trials and all blocks for each participant and then comparing data between trials with error lower than the median (good trials) to those with error greater than the median (bad trials). To be included in the performance analysis, three or more artefact free good and bad trials on at least one channel were required. For phase‐amplitude coupling (PAC) analyses, these results were confirmed by splitting each patient's trials into terciles according to their distance error.

*Time–Frequency Analysis*. Estimates of dynamic oscillatory power during periods of interest were obtained by convolving the EEG signal with a five‐cycle Morlet wavelet. Time–frequency data were extracted from 5 s before the start of each 3 s cue period to 2 s after the end of that period. Power values were then obtained for 30 logarithmically spaced frequency bands in the 2–110 Hz range, log transformed, and the mean power in each frequency band during a 2 s baseline period immediately preceding the presentation of the cue were subtracted from data at each time point during the 3 s cue period, to give measures of power change in each frequency band from baseline. Data from time windows before the baseline period and after the cue period were discarded after convolution to avoid edge effects. All trials that had visually identified interictal spikes or other artefacts (such as electrical noise or brief disconnection of recording, occurring in a total of three trials within two patient recordings), either within the period of interest or during the padding windows, were excluded from all analyses presented here (Table 1). Each patient performed either one or two blocks, each consisting of 20 trials, providing a mean ± *SD* of 19.6 ± 11.9 trials for analysis after artefact rejection.

**FIGURE 2:**
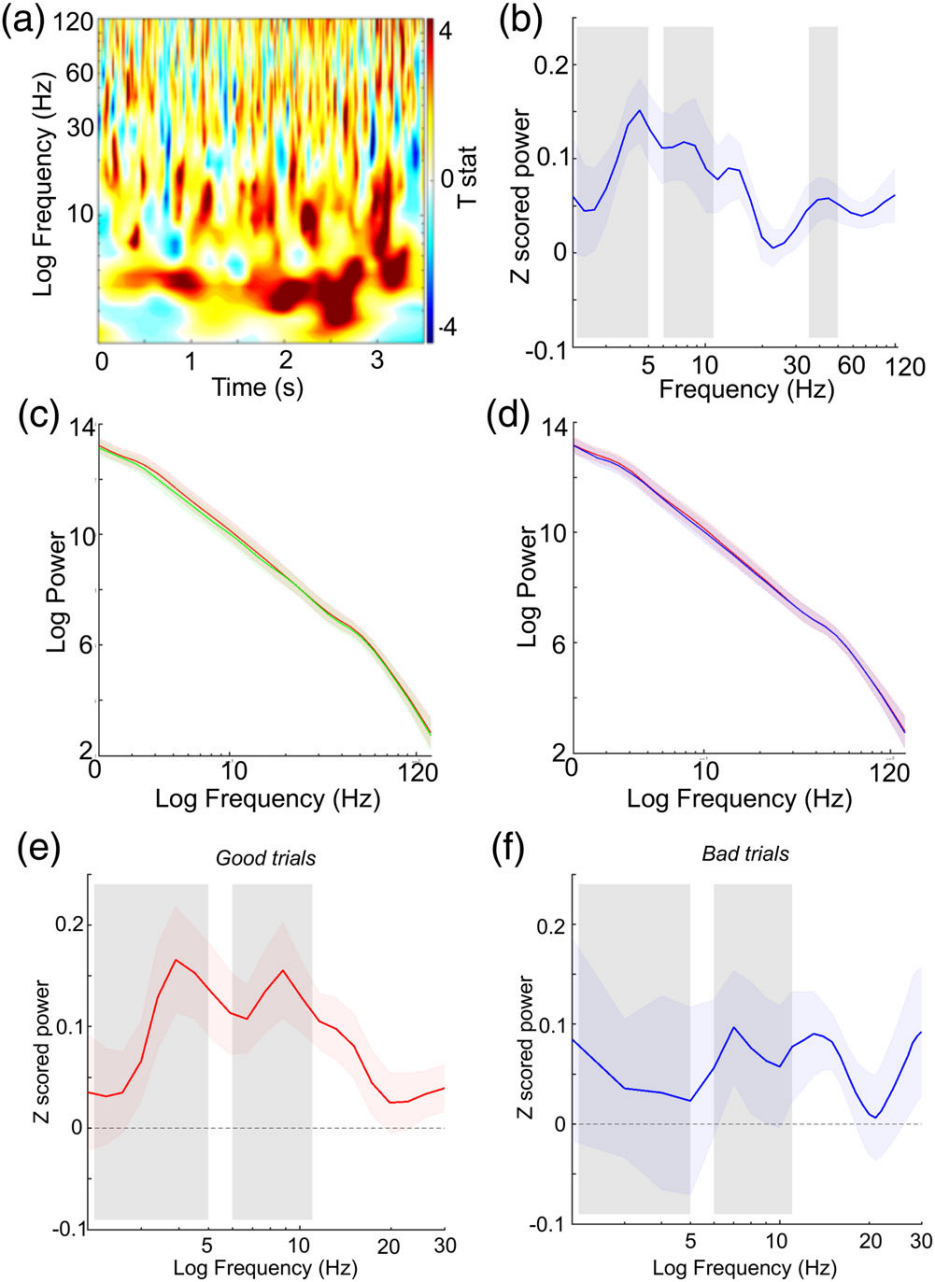
Retrieval is associated with increased power within specific frequency bands and low and high theta power in the hippocampus are associated with improved performance. (a). Time‐frequency power spectrogram for all trials during the cue period at group level. (b). Baseline corrected cue period power spectra at group level demonstrating significant clusters in low (2–5 Hz) and high (6–11 Hz) theta bands, and low (35‐50 Hz) gamma band, marked with shaded boxes. (c). Log power during cue (red) and baseline (green) periods at group level demonstrating no significant change in spectral tilt; shaded outline represents SEM. (d). Log power across the cue period during good trials (red) and bad trials (blue) at group level. (e). Mean baseline corrected power in the hippocampus during cue periods of good‐performance trials at group level, with low (2–5 Hz) and high (6–11 Hz) theta bands marked with shaded boxes. (f). Mean baseline corrected power in the hippocampus during cue periods of bad‐performance trials [Color figure can be viewed at wileyonlinelibrary.com]

Changes in log‐transformed oscillatory power from baseline were averaged across the whole 3 s cue period and all electrode contacts for each subject, then subsequently analysed using a Monte Carlo cluster permutation approach in order to find clusters in frequency space. The permutation test consists of two steps: first, clusters of coherent *t*‐values exceeding a certain threshold (corresponding to *p* < .05) along the frequency dimension are detected in the data; and second, summed *t*‐values for these clusters are compared to a null distribution of summed *t*‐values for random clusters obtained by permuting condition labels across subjects. Mean power values within each trial were subsequently calculated for each frequency band, averaged across contacts for each patient, and then analysed using one sample *t*‐tests (baseline corrected cue period versus zero) or repeated‐measures ANOVAs and post hoc one‐sample *t*‐tests, both at the subject level (see Figure 2b).

To establish whether within‐subject changes in low and high theta power across trials were associated with performance, we performed a linear regression between theta power and distance error across trials separately for each electrode contact. Beta coefficients were then averaged across all electrode contacts for each patient, allowing one‐sample *t*‐tests to be performed at the second level. To examine evidence of spectral tilt or spectral shift, we generated time‐averaged log‐transformed power spectra separately for the baseline and nonbaseline corrected cue periods, as well as nonbaseline corrected cue periods in good versus bad trials, for each electrode contact. We then performed linear regression analysis of log frequency versus power to acquire slope (i.e., tilt) and intercept (i.e., shift) values for each electrode contact, averaged those values across all electrode contacts for each patient, and compared spectral tilt and shift between conditions using one‐sample *t*‐tests.

**FIGURE 3:**
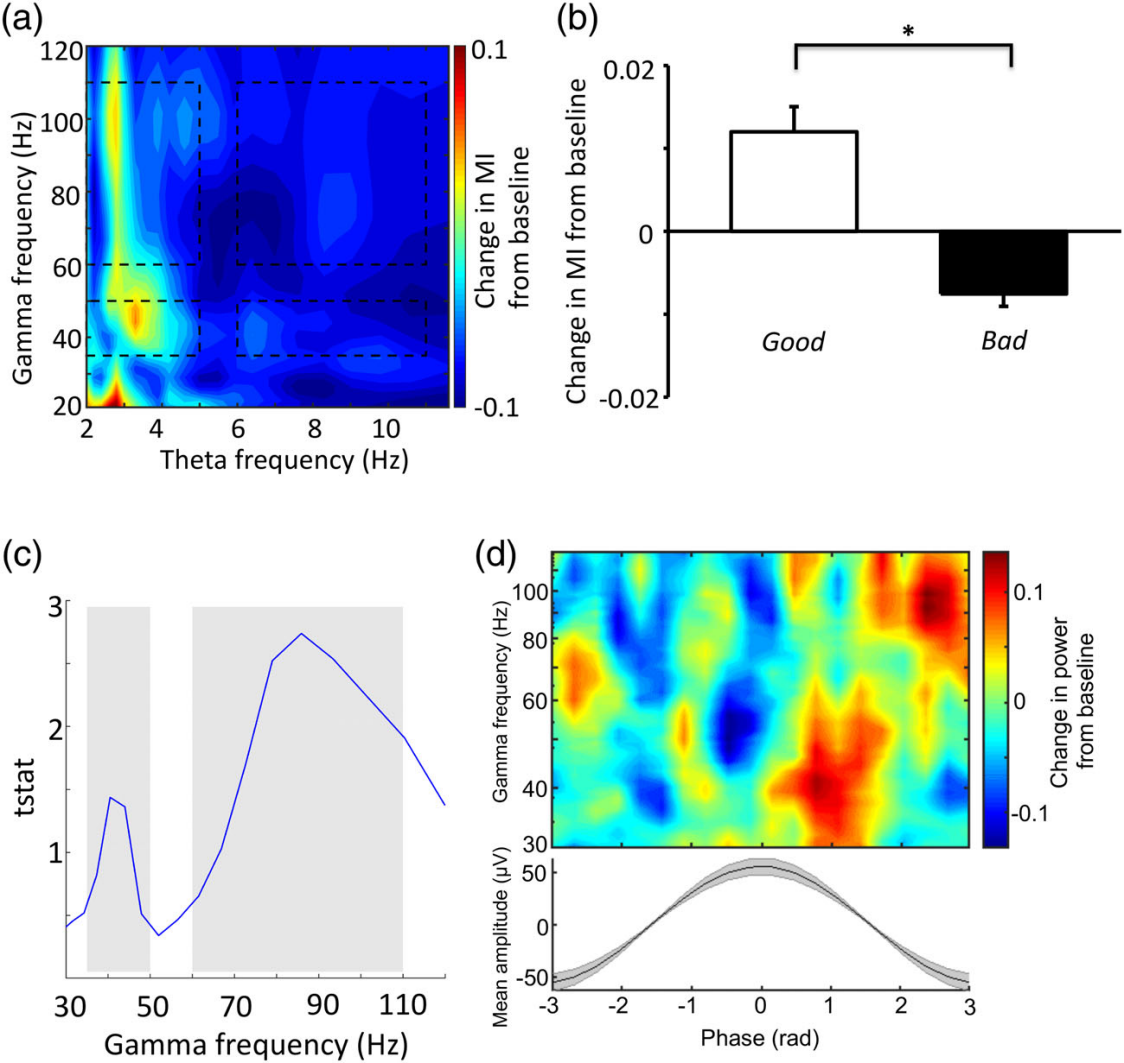
Increased low theta phase modulation of gamma amplitude is associated with improved performance. (a). Cross frequency spectrogram showing contrast of good‐bad performance trials at group level; boxed regions highlight potential regions of coupling between 2–5 Hz low and 6–11 Hz high theta phase and 35–50 Hz low and 60–110 Hz high gamma amplitude. (b). Mean change in modulation index values from baseline between low theta phase and low and high gamma for good performance (white), and bad performance (black) trials at group level. Modulation index values between low theta phase and amplitude in each gamma band are averaged for each electrode contact, before being averaged across electrode contacts for each patient. Error bars are SEM. (c). T‐statistics for modulation index between low theta and the full range of gamma frequencies, during good trials. (d). Average phase frequency spectrogram for theta phase and z‐scored gamma power (top) and average theta cycle (bottom) at group level [Color figure can be viewed at wileyonlinelibrary.com]

*Phase Amplitude Coupling*: Next, we examined PAC between the phase of two theta frequency bands (2–5 Hz and 6–11 Hz) and the amplitude of two gamma bands on the same electrode contacts (35–50 Hz and 60–110 Hz; following (Colgin et al., 2009). Phase‐amplitude coupling PAC was estimated using the modulation index (Tort et al., 2009). Modulation Index employs all possible phases from −180 to 180°, which are first binned into an optional amount of bins, and the average amplitude of the amplitude‐providing frequency is subsequently computed and normalised for each phase bin. PAC is then defined by a distribution that significantly deviates from the uniform distribution. To avoid reporting PAC with a different preferred phase in each trial, phase and amplitude data were concatenated over all trials before computing a single modulation index value for each contact. Modulation index values were then averaged over all electrode contacts for each patient, and comparisons were made at the subject level using repeated measures ANOVAs. For illustrative purposes, we also computed modulation index values across a range of low frequency phases (in the 2–12 Hz range) and high frequency amplitudes (in the 20–120 Hz range, see Figure 3a,b, Supplementary Figure 1), although these data were not used for any statistical analyses. In order to confirm that the increased modulation index observed during good trials (i.e., those with below median distance error) was not due to chance, we performed 100 random shuffles of phase and amplitude data (shifting the amplitude data relative to the phase data by at least 100 samples, that is, ~200 ms) and then calculated the mean modulation index value across all shuffles for each electrode contact, before averaging over electrode contacts to obtain a shuffled baseline modulation index value for each pair of (phase and amplitude) frequency bands for each patient.

In addition, to establish whether observed changes in PAC according to performance resulted purely from changes in the signal to noise ratio of low frequency power, we performed linear regression between modulation index values (computed separately for each trial) and theta power. Beta coefficients for modulation index versus low theta power were obtained, which were averaged across all electrode contacts for each patient allowing one‐sample *t*‐tests to be performed.

Finally, we estimated the preferred theta phase of power in each gamma band by extracting the (weighted) circular mean of low and high gamma power distributions across 20 theta phase bins separately for each electrode contact, computed as part of the modulation index pipeline outlined above. We then obtained the circular mean preferred theta phase across electrode contacts for each patient, and preferred theta phase distributions for low and high gamma power across patients were compared using a parametric Watson–Williams multisample test (Circular Statistics Toolbox;(Berens, 2009). For illustrative purposes, we also used the time frequency representation generated for each trial and electrode contact to compute average power in each theta phase bin across a range of gamma frequencies (see Figure 3e). Power values at each frequency were then normalised by average power across all theta phase bins, averaged across electrode contacts for each patient, and then across patients (although these data were not used for any statistical analyses).

In order to examine correlations between low and high gamma across theta cycles, we performed linear regression analysis of low gamma versus high gamma power during every theta cycle within each cue period across all trials for each hippocampal contact. We then averaged beta coefficients across electrode contacts for each patient, and assessed the resultant distribution using a one‐sample *t*‐test.

## 3 RESULTS

### 3.1 Object location task performance correlates with formal assessment of spatial memory

Our long‐term spatial memory task involved patients first navigating toward and memorizing the location of four objects that sequentially appeared in the environment (“encoding”). Patients were then cued with an image of one object (“cue”), placed back in the environment and asked to navigate toward the remembered location of that object and make a button‐press (“response”). The object then appeared in its correct location and the trial ended when they moved to the visible object (“feedback”) (Figure 1a). Performance contrasts were computed by taking the median distance error across all trials for each participant. Prior to this study, all patients underwent formal neuropsychometry testing as preclinical evaluation for epilepsy surgery. As part of that testing, all patients (apart from one) were asked to perform a complex figure recall task from the BIRT Memory and Information Processing Battery (coughlan et al., 2007), as a measure of visuospatial memory, and an accuracy score was given for performance. We correlated patient performance during neuropsychometry testing with their median distance error during our object location task. We found that there was a significant negative correlation between immediate recall of the complex figure and distance error in our task (Pearson's r = −.71, *p* = .032; Figure 1b), indicating some relationship between our measure of spatial memory and a widely used clinical measure of visuospatial memory performance.

### 3.2 Increased theta and gamma power in hippocampus during retrieval

Next, we examined changes in oscillatory power on hippocampal electrode contacts averaged across the whole 3 s cue period, when participants were asked to retrieve the location of an object prior to being randomly replaced in the virtual environment (Figure 2a). To identify frequency bands of interest we used data driven Monte Carlo cluster analysis. We found significant positive clusters in low (2–5 Hz; *t*_sum_ = 12.0, *p* < .001) and high (6–11 Hz; *t*_sum_ = 8.05, *p* = .007) theta and low gamma (35–50 Hz, *t*_sum_ = 4.78, *p* = .013) frequency bands during the cue period compared to baseline (see Methods). These clusters were confirmed when examining baseline corrected power versus frequency during retrieval cue periods (Figure 2b). This suggests that low and high theta and low gamma oscillations in the hippocampus are engaged by the retrieval of long‐term spatial memories. Despite the observed increases in both low (theta) and high (gamma) frequency power, we found no change in the slope (i.e., spectral tilt) (*p* = .59) or intercept (i.e., spectral shift) (*p* = .431; Figure 2c) of power spectra between baseline and cue periods. This is consistent with increases in narrowband (theta and gamma) power, without a change in the overall profile of the power spectrum.

### 3.3 Increased theta power in hippocampus during retrieval predicts performance

Next, in order to establish whether increased theta and / or gamma power in the hippocampus were associated with task performance, we conducted a repeated measures ANOVA with factors of performance (good v bad trials) and theta frequency band (low v high). We found a significant effect of performance (F(1,9) = 6.4, *p* = .035), but no interaction (*p* = .68), driven by increased low and high theta power during good trials (Figure 2e,f). Repeating this analysis with factors of performance (good v bad) and low gamma frequency band revealed no significant effect of performance (*p* > .07). Consistent with these results, linear regression between low and high theta power and performance on a trial by trial basis (see Methods) revealed a significant negative relationship between both low (t(9) = 2.85, *p* = .01) and high (t(9) = 2.29, *p* = .047) theta power and distance error.

Finally, it has been suggested that successful memory formation is associated with “spectral tilt” (i.e., concomitant increases / decreases in low / high frequency activity, characterised by a change in the slope of the power spectrum), or spectral “shift” (i.e., concomitant increases in low and high frequency activity) (Burke et al., 2013). To examine whether this might also be true for memory retrieval, we performed linear regression analysis between log frequency and log power during cue periods from good and bad trials, but found no significant difference in spectral tilt (*p* = .12) or shift (*p* = .303) with task performance (Figure 2d). This suggests that increased theta power within the hippocampus during the cue period is associated with accurate performance of the object location task, without any significant change in overall spectral “tilt” or “shift.”

### 3.4 Increased theta‐gamma phase amplitude coupling in hippocampus during retrieval predicts performance

To further dissect the role of low and high theta oscillations in spatial memory retrieval, we examined changes in phase‐amplitude coupling (PAC) between low or high frequency theta phase and gamma amplitude using the modulation index (Tort et al., 2009). First, we conducted a repeated measures ANOVA for all trials with factors of theta frequency band (low v high), gamma frequency band (low v high) and cue versus baseline periods, but found no significant differences in PAC according to these factors (all *p* > .11). In addition, modulation index values between any pair of theta (2–5 Hz and 6–11 Hz) and gamma (low 35–50 Hz and high 60–110 Hz) frequency bands were not significantly different from zero (all *p* > .059), suggesting that there was no overall change in theta‐gamma PAC during spatial memory retrieval.

Next, to examine if PAC in the hippocampus was relevant for task performance we performed a three way ANOVA with factors of performance (good v bad), theta frequency band (low vs high) and gamma band (35–50 Hz vs 60–110 Hz) in nine patients that had sufficient artefact free trials for analysis. We found that modulation index values differed significantly between good and bad performance trials (F(1,8) = 8.31, *p* = .02), with a significant interaction between good/bad trials and low/high theta (F(1,8) = 6.96, *p* = .03), but no other interactions (*p* > .17). Subsequent analysis indicated that these results were driven by increased modulation of both low and high gamma amplitude by the phase of low theta band oscillations during good trials (*t*(8) = 2.63 *p* = .03; Figure 3a,b). Importantly, this difference in modulation index values persisted when dividing trial performance into terciles, and comparing modulation index values in the top and bottom tercile, rather than using a median split (t(8) = 2.76 *p* = .024; Supplemental Figure 1). To establish whether these results were driven by an increase in PAC during good performance trials, a decrease in PAC during bad performance trials, or both, we next compared mean modulation index values during good and bad performance trials with the baseline period. We found that modulation index values associated with good performance trials were significantly higher than baseline (t(8) = 4.54 *p* = .002) and modulation index values associated with bad performance trials were not significantly lower than baseline (*p* = .07). Furthermore, we found that modulation index values within good performance trials were significantly higher than that observed after randomly shuffling the phase/amplitude data (t(8) = 2.31 *p* = .039). This indicates that increased low‐theta gamma PAC in the hippocampus is associated with successful spatial memory retrieval.

Next, in order to ascertain whether the increase in hippocampal low theta‐gamma PAC during good performance trials solely resulted from the increase in low theta power (and therefore signal‐to‐noise ratio) described earlier, we performed a regression analysis between modulation index values and low theta power. We found no evidence for a significant linear relationship between low theta power and modulation index values across trials (*p* > .51). This suggests that increased low theta phase modulation of both low and high gamma amplitude in the hippocampus is associated with improved task performance, independent of concurrent changes in low theta power.

Finally, we examined the preferred low theta phase of low and high gamma oscillations during cue periods. Previous rodent studies have demonstrated that increases in CA1 low and high gamma power occur at different theta phases, associated with input from CA3 and entorhinal cortex, respectively (Colgin et al., 2009). We also found that peak low and high gamma power were associated with different phases of theta (Figure 3d). Low gamma power was maximal around the peak of theta (0–90°) and high gamma power was maximal on the downward slope of theta (90–180°) (Supplemental Figure 2). Importantly, the preferred phase of low and high gamma differed significantly (F(2,19) = 4.52, *p* = .047; parametric Watson‐Williams multisample test for equal means). However, unlike rodent data (Colgin et al., 2009), where low and high gamma oscillations tended to occur in different theta cycles, we observed a significant correlation between low and high gamma power across theta cycles during retrieval cue periods (t(9) = 5.91, *p* < .001), suggesting that low and high gamma power tended to co‐occur in the human hippocampus.

## 4 DISCUSSION

The medial temporal lobe plays a crucial role in both spatial navigation and memory function. In humans, the hippocampal theta rhythm appears to make a functional contribution to both processes, with increased theta power and mesial temporal connectivity observed during episodic memory formation (Lega et al., 2012). Separate low (2‐5 Hz) and high (6‐11 Hz) theta bands have been observed during short‐term mnemonic function (Lega et al., 2012; Watrous et al., 2013) in humans, but their specific role in long‐term spatial memory function has remained unclear. Here, we have demonstrated distinct roles for low and high theta within the hippocampus during the performance of a long‐term spatial memory task. Specifically, during spatial memory retrieval (i.e., during the cue periods of our object location task), both low and high theta power and low gamma power are increased in the hippocampus. We had previously shown that hippocampal theta power was indicative of movement onset in this task (Bush et al., 2017) and spatial memory retrieval in MEG (Kaplan et al., 2014). Other studies performing a different hybrid navigation and memory paradigm have suggested diverse roles for low theta depending on laterality and hippocampal subregion (Miller et al., 2018; Goyal et al., 2020; Herweg et al., 2020). There has been an increasing body of evidence to suggest that low frequency power decrease and high frequency power increase is associated with performance in a variety of tasks, with this “tilt” in the 1/frequency characteristic inherent to human EEG thought to reflect increased regional neural activity (Fellner et al., 2019). However, we found that low and high theta power increases alone after correction for multiple comparisons were associated with task performance, as previously reported in both MEG (Kaplan et al., 2012) and intracranial EEG (Miller et al., 2018) studies. These results are more consistent with a “spectral fingerprint” framework, which suggests that oscillatory changes at discrete frequency bands reflect different neural processes.

In humans, hippocampal broadband frequency phase clustering has been demonstrated during successful word recognition (Mormann et al., 2005), and increased frontal theta phase modulation of posterior gamma amplitude coupling during visual encoding (Friese et al., 2013). Our finding of performance related increases in the hippocampal modulation of low (35–50 Hz) and high gamma (60–110 Hz) amplitude by low theta phase after correction for multiple comparisons extends existing findings to the domain of long term spatial memory retrieval. The validity of PAC in certain scenarios has been questioned recently, especially for nonsinusoidal low frequency oscillations (Kramer et al., 2008). However, the use of the Modulation Index to estimate PAC is less sensitive to signal to noise issues. In addition, the presence of two discrete gamma bands visible in the local field potential argues that the findings reported here are reliable (Jensen et al., 2017).

Rodent models suggest that these gamma frequency bands mediate the routing and temporal segregation of inputs to the hippocampal CA1 region from different sources (Colgin et al., 2009). Consistent with those data, we have demonstrated that the power of low and high gamma oscillations have different preferred low theta phases (although not corrected for multiple comparisons), and are therefore temporally segregated within each low frequency cycle. Such phase coding of information has been previously documented in human ECoG studies (Watrous et al., 2015), although without any relationship to performance. Indeed, more recent studies have described the preferred phase of firing for individual neurons during virtual navigation (Watrous et al., 2018) and future work could investigate the role of phase coding at a single neuron level in long term spatial memory.

In summary, consistent with a growing body of literature, we have demonstrated that theta band oscillations are functionally relevant to long‐term spatial memory retrieval, while low theta‐gamma phase amplitude coupling has a role in accurate spatial memory performance. Limitations of the study included an insufficient number of patients to properly address laterality and regionality of hippocampal theta activity, and the absence of a distractor task in between encoding and retrieval phases to avoid any contribution of short‐term memory processes. Future work should examine the specific contribution of low and high gamma oscillations to task performance, and whether these reflect afferent inputs from, or output targeted at, different cortical regions connected to the hippocampus.

## Supporting information


**Supplemental Figure 1** **Increased low theta phase modulation of gamma amplitude remains associated with improved performance when trials are divided into terciles**. Cross frequency spectrogram showing contrast of top tercile‐bottom tercile performance trials at group level; boxed regions highlight potential regions of coupling between 2‐5 Hz low and 6‐11 Hz high theta phase and 35‐50 Hz low and 60‐110 Hz high gamma amplitude.

**Supplemental Figure 2** A. **Example raw trace** from one cue period showing theta, low gamma and high gamma waveforms. B. Polar plots of preferred low theta phase for low gamma amplitude (35‐50 Hz) and C. High gamma amplitude (60‐110 Hz). In each case, average gamma power was computed for each theta phase bin across all trials on each electrode contact, normalised by total power summed across theta phase bins, then averaged across electrode contacts for each patient. Data shown are group level averages across patients.

## Data Availability

Anonymized data can be shared by request from any qualified investigator
