# Supplementary figures and images for: Theta power and theta‐gamma coupling support long‐term spatial memory retrieval
Source: Hippocampus. 2020 Dec 2;31(2):213–20. doi: 10.1002/hipo.23284 (PMC7898809; doi:10.1002/hipo.23284)

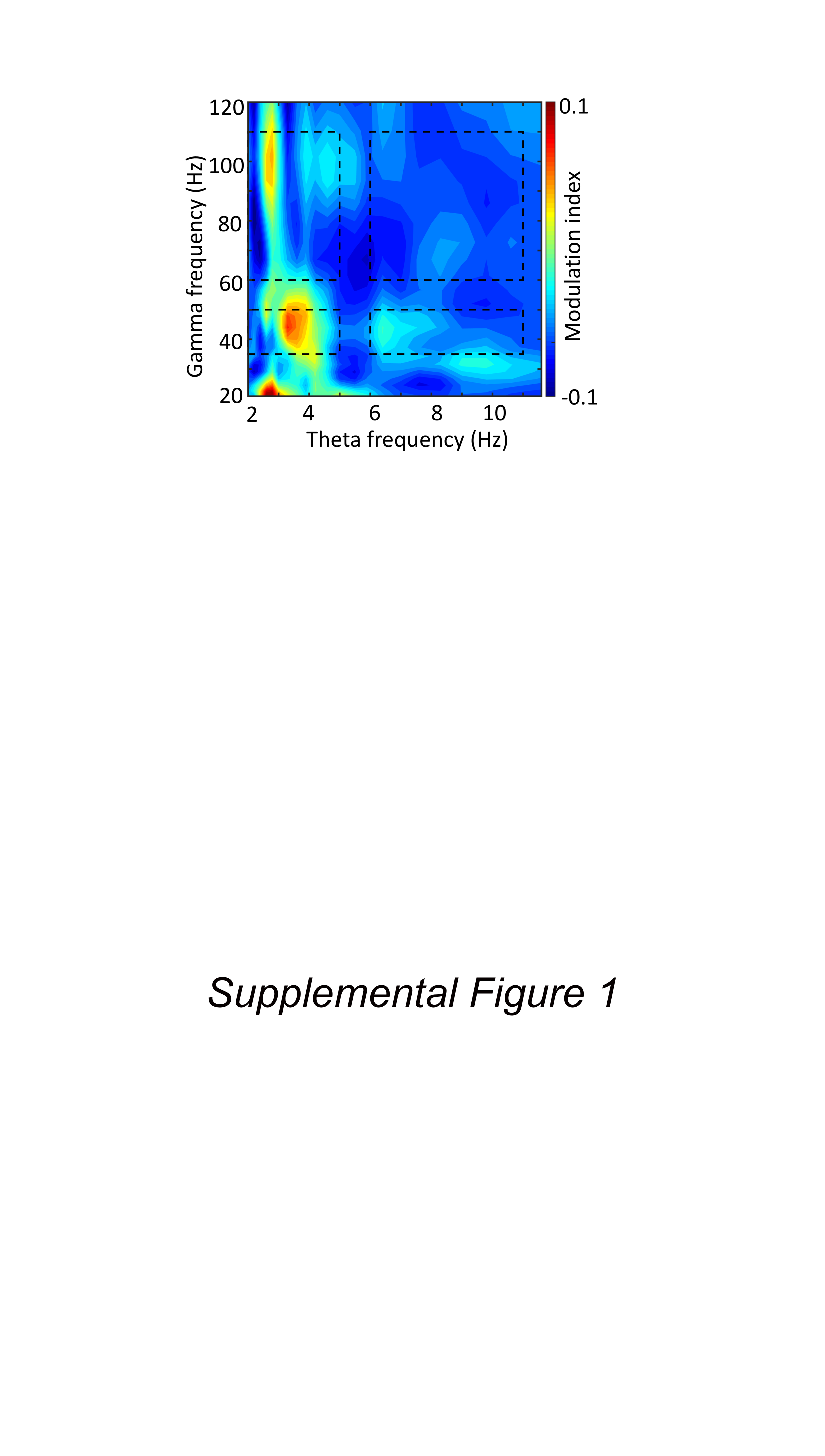

Supplement: Supplementary file 1 — Supplemental Figure 1 Increased low theta phase modulation of gamma amplitude remains associated with improved performance when trials are divided into terciles. Cross frequency spectrogram showing contrast of top tercile‐bottom tercile performance trials at group level; boxed regions highlight potential regions of coupling between 2‐5 Hz low and 6‐11 Hz high theta phase and 35‐50 Hz low and 60‐110 Hz high gamma amplitude. [file HIPO-31-213-s001.tif]

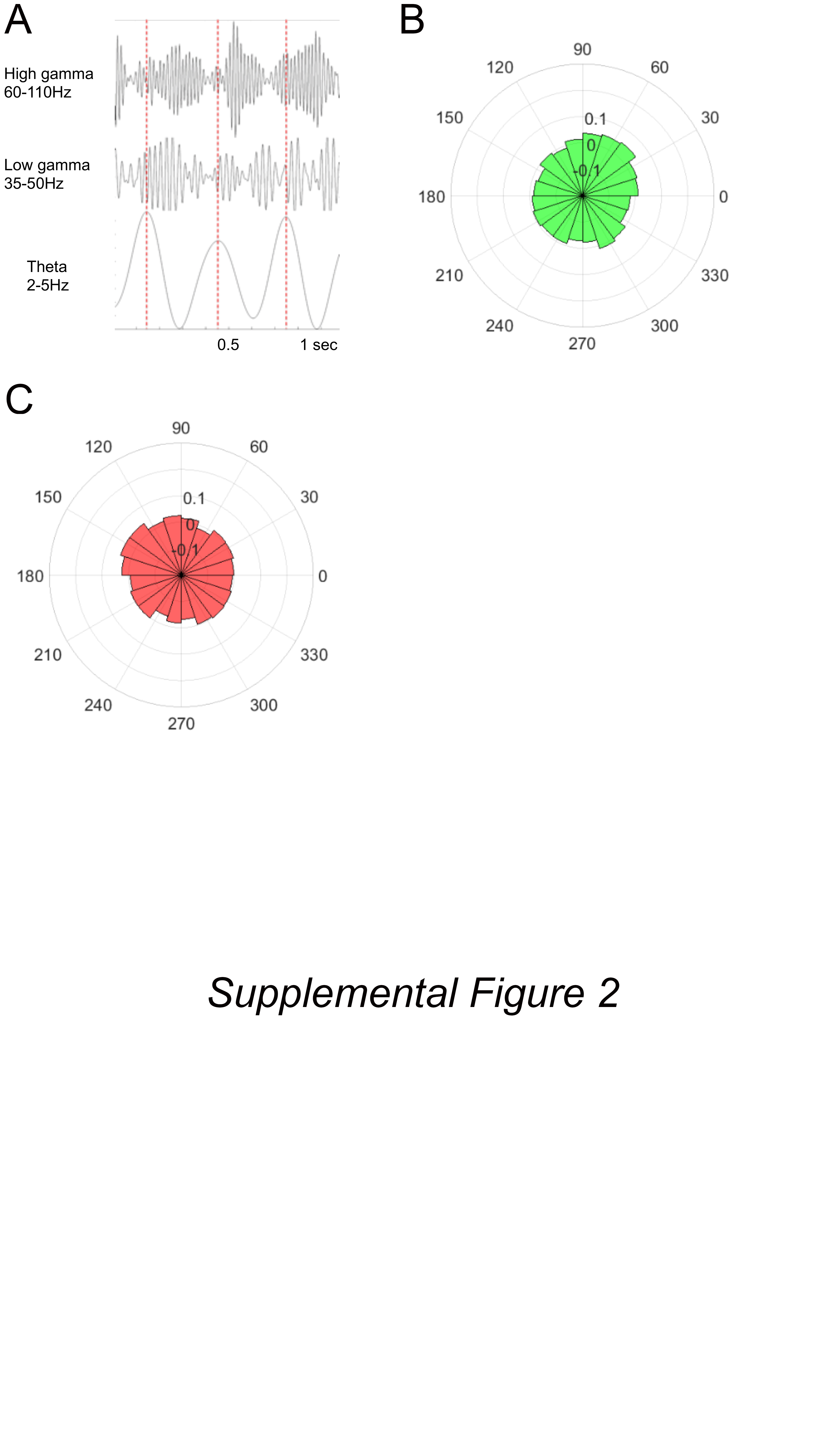

Supplement: Supplementary file 2 — Supplemental Figure 2 A. Example raw trace from one cue period showing theta, low gamma and high gamma waveforms. B. Polar plots of preferred low theta phase for low gamma amplitude (35‐50 Hz) and C. High gamma amplitude (60‐110 Hz). In each case, average gamma power was computed for each theta phase bin across all trials on each electrode contact, normalised by total power summed across theta phase bins, then averaged across electrode contacts for each patient. Data shown are group level averages across patients. [file HIPO-31-213-s002.tif]
